# Social barriers in accessing care by clients who returned to HIV care after transient loss to follow-up

**DOI:** 10.1186/s12981-019-0231-5

**Published:** 2019-08-12

**Authors:** Babatunde Adelekan, Nifarta Andrew, Iboro Nta, Asabe Gomwalk, Nicaise Ndembi, Charles Mensah, Patrick Dakum, Ahmad Aliyu

**Affiliations:** 1 Institute of Human Virology, Nigeria (IHVN), Abuja, Nigeria; 2 Present Address: Academy for Health and Development, Ile-Ife, Nigeria

**Keywords:** Loss to follow-up (LTFU), Anti-retroviral therapy (ART), Social barriers, HIV, PLHIV, Nigeria

## Abstract

**Background:**

People living with HIV (PLHIV) constantly need to address social issues such as the cost of accessing care, stigma, and lack of social support which impacts on their level of adherence to clinic visits or antiretroviral treatment leading to adverse health outcomes. This study examined the social barriers in accessing care by clients who returned to care after transient loss to follow-up.

**Methods:**

This study was a cross-sectional survey of PLHIV from 99 US CDC PEPFAR-supported HIV clinics located in 10 of Nigeria’s 36 states and Federal Capital Territory, who were momentarily lost to follow-up but returned to care after tracking. Demographic and social factors at bivariate and multivariate level were analyzed to determine the predictors of difficulty in accessing HIV clinics.

**Results:**

Of the 7483 clients tracked, 1386 (18.5%) were confirmed to be in care, 2846 (38.2%) were lost to follow-up (LTFU), 562 (7.5%) returned to care, 843 (11.2%) discontinued care, 827 (11.1%) transferred out to other facilities for care, 514 (6.8%) had died while 505 (6.7%) could not be reached by phone or located at their addresses. 438 out of the 562 (78%) returnee PLHIV gave consent and participated in the study. 216 out of the 438 (50%) clients who returned to care were transiently lost to follow-up because they had difficulty accessing their HIV clinic. Also, 126/438 (29%) of returnee PLHIV were previously lost to follow-up. Difficult access to a HIV clinic was significantly influenced by prior LTFU (OR 2.5 [95% CI 1.3–4.8], p = 0.008), history of being stigmatized (OR 2.1 [95% CI 1.1–3.8], p = 0.02), lack of social or financial support (OR 2.8 [95% CI 1.3–6.0], p = 0.01) and perceived in-adequate healthcare workers support (OR 3.8 [95% CI 1.2–11.2], p = 0.02). Age (p = 0.218) and gender (p = 0.771) were not significant determinants of difficult access to an HIV clinic.

**Conclusion:**

Stigma, lack of support and prior loss to follow-up event are essential factors affecting retention in care. Social constructs such as home-based visits, community-based care services, transportation subsidies, and robust strong social systems should be built into HIV service delivery models to improve retention in care of people on HIV treatment. The authors advocate for further studies on how differentiated care models impact on retention of patients in care.

## Background

In the past decade, improved access to antiretroviral therapy (ART) worldwide has been remarkable, yet failure to retain HIV-infected individuals in treatment programs due to losses to follow-up (LTFU) is common [1] in many low and middle-income countries [2, 3] including Nigeria [4–7] and the sub-Saharan African region (sSA) [8]. Program studies in Nigeria typically report above 20% LTFU at greater than 12 months [4, 5].

LTFU impacts epidemic control by reducing retention across the care-continuum thus limiting optimum health outcomes [9] for PLHIV and distorting global evidence for the preferred outcome of HIV treatment in an era of rapid ART scale-up [10, 11]. A myriad of factors impact LTFU are well documented including those related to poverty e.g. lack of food or transportation, poor health communication, such as non-disclosure of HIV status, health systems inefficiencies such as drug stock-outs [12–14]. For example, the primary determinant for disrupting ART for 57% of confirmed LTFUs among PLHIVs in a Swaziland report was hunger and no transit funds to reach the health facility [15].

People living with HIV (PLHIV), frequently contend with psychological and social issues which may affect their adherence to clinic visits and ART [16, 17]. In these settings, apart from coping with the primary fears and distress of living with a life-threatening disease, discrimination and stigmatizing behavior [18] complicates their successful management. However, psychosocial predictors of LTFU have been assessed less frequently, perhaps due to the relative difficulty of obtaining this information [19].

We examined the opinions and experiences of HIV infected clients on ART who were transiently LTFU but returned to care and explored factors associated with their difficulty in accessing HIV clinics. Improvement in this aspect of the care cascade derived from proper studies may positively impact program effectiveness and efficiency in low middle-income countries (LMICs) [10, 20, 21].

## Methodology

### Study design and settings

This cross-sectional survey was conducted in 3 weeks between March and April 2017 in 99 Health Care Facilities (HCF) spread across 10 of Nigeria’s 36 states and Federal Capital Territory. These HCF have a HIV/AIDS program that is supported by the United States President’s Emergency Plan for AIDS Relief (PEPFAR). Adult PLHIV placed on ARV who were transiently LTFU were identified using an electronic medical record systems (EMRS) generated list. PLHIV were considered LTFU according to PEPFAR-Nigeria’s supported program’s definition of 90 days after a missed clinical and or pharmacy pick-up appointment.

The Institute of Human Virology Nigeria (IHVN), a non-Governmental Organization that partners with several Nigerian states to provide quality HIV services collaborated with the Network of People Living with HIV and AIDS in Nigeria (NEPWHAN) to improve tracking and return to care transiently LTFU PLHIV. A master list by State of presumed LTFU clients generated from all participating HCF was sent to NEPWHAN which then utilized State teams to track down clients.

NEPWHAN, Nigeria’s foremost and largest PLHIV-run civil society organization [22], with a nationwide spread of well-trained PLHIV counsellors and volunteers tracked identified clients using pre-consented contact information-phone calls or home addresses, to ascertain their status and encourage a return to care. A standard program questionnaire was administered to PLHIVs who returned to care. The structured questionnaire had sections that covered demographics, relationships and support, stigma and disclosure, access to care (financial and geographical) and reasons for being transiently lost to follow-up. The study was approved by the institution’s (Institute of Human Virology, Nigeria) Research Ethics Committee and is covered by the NHREC approval for program evaluation. Informed consent of clients was obtained before the re-entry in care interviews.

### Data collection and analysis

Data was entered in Microsoft Excel, imported, cleaned and analyzed with SPSS version 20 (BM Corp. Released 2011. IBM SPSS Statistics for Windows, Version 20.0. Armonk, NY: IBM Corp.). Proportions were calculated for all variables. Bivariate analysis was conducted using, Chi-square tests while multivariate analysis was conducted through logistic regression with p < 0.05 considered statistically significant.

## Results

Of the 7483 clients tracked, 1386 (18.5%) were confirmed to be in care, 2846 (38.2%) were lost to follow-up (LTFU), 562 (7.5%) returned to care, 843 (11.2%) voluntarily discontinued care on a claim of being healed, 827 (11.1%) transferred out to other facilities for care, 514 (6.8%) had died while 505 (6.7%) could not be reached by phone or located at their addresses. 438 of 562 (77.9%) agreed to participate in the review.

Returnee PLHIV had a median age of 36 years (IQR 56, SD 9.8), 66% were married and 67.4% were female. Most (32%) had their diagnosis within 2 years of this study and had median times on ARV and cotrimoxazole prophylaxis of 2 (IQR 16, SD 3) and 3 (IQR 17, SD 3) years respectively (not shown). Formal education was common among study participants with 67.8% of returnee PLHIV, and 60% of their intimate partners going beyond primary school.

About half (49.31%) of returnee PLHIV reported that access to an HIV clinic was difficult while a third (28.8%) of the returnee PLHIV had a previous LTFU event.

HIV sero-discordant rate was 30.6% while 88.4% of returnee PLHIV had disclosed their HIV status to family members (44.1%) and spouses (34.7%), compared to others (friends and faith advisers). Just about a quarter of them (23.5%), reported experiencing or suspecting stigmatizing behavior. Community and neighbors (8.5%), family members (7.3%) and friends (6.2%) were alleged to stigmatize the most, while spouses and healthcare workers the least. Support from the family was robust for 82.7% respondents (not shown), but 54.1% complained of low financial support and 19.9% signified need for emotional/psychological support (see Table 1).

**Table 1:** Frequency table of demographic and social related factors of patients

Demographic characteristics	Total (n = 438) n (%)	Social related factors	Total (n = 438) n (%)
Sex		Person status disclosed to	
Female	295 (67.35)	Partner/spouse	152 (34.70)
Male	137 (31.28)	Family	193 (44.1)
Missing	6 (1.37)	Other	35 (8.00)
Religion		Nobody	51 (11.00)
Muslim	137 (31.28)	Missing	7 (1.60)
Christian	275 (62.79)	Disclosed status	
Traditional/others	10 (2.28)	Yes	380 (88.40)
Missing	16 (3.65)	No	51 (11.00)
Relationship status		Missing	7 (1.60)
Married	289 (65.98)	Partner status	
Single	70 (15.98)	Know positive	176 (40.18)
Divorced/separated	35 (7.99)	Know negative	134 (30.60)
Widowed	44 (10.05)	Don’t know	82 (18.72)
Educational status		Missing	46 (10.50)
None	4 (0.91)	Is access to facility difficult	
Primary	110 (25.11)	Yes	216 (49.31)
Secondary	177 (40.41)	No	78 (17.81)
Post-secondary	120 (27.40)	Missing	144 (32.88)
Missing	27 (6.16)	Prior LTFU	
Educational status of partner		Yes	126 (28.77)
None	0 (0)	No	300 (68.49)
Primary	91 (20.78)	Missing	12 (2.74)
Secondary	140 (31.96)	Stigma	
Post-secondary	123 (28.08)	Yes	103 (23.52)
Missing	41 (9.36)	No	323 (73.74)
Age		Missing	12 (2.74)
< 24	30 (6.85)	Type of lack of support	
25–35	170 (38.81)	Financial	237 (54.11)
36–46	137 (31.28)	Psychological/emotional	87 (19.86)
> 47	71 (16.21)	Other	77 (17.58)
Missing	30 (6.85)	Missing	37 (8.45)
Duration since HIV status is known		Stigma by whom	
< 1 year	42 (9.59)	Community/neighborhood	37 (8.45)
1–2 years	140 (31.96)	Family	32 (7.31)
3–5 years	127 (29.00)	Friend	27 (6.16)
> 5 years	126 (28.77)	Healthcare worker	15 (3.42)
Missing	3 (0.68)	Partner/spouse	14 (3.20)
		Missing	313 (71.46)

The associations between demographic related factors and access to facility were not significant (sex; p = 0.771, age; p = 0.218, religion; p = 0.503, relationship status; p = 0.874, educational status; p = 0.793, educational status of partner, p = 0.385, duration since HIV status is known; p = 0.782). However, transiently lost to follow-up clients who returned to care that are females (73.9%), widows (77.4%), between the age 36–46 years (80.6%), who practiced traditional and other religion apart from Christianity and Islam (88.8%), without any formal education (100%) and whose partners had other educational status other than those indicated (75.9%) and have known their HIV status for 3–5 years (77.8%) have difficulties in accessing their facilities (not shown).

Previous history of being LTFU is associated with difficulty in accessing the health facility (p = 0.03). Bad/dangerous roads, cost of transportation, far distance to the facility and long working hours are all strongly associated with difficulty in accessing the health facility (p < 0.001).

Clients had a justification for choosing the facilities including access to female providers, availability of drugs, proximity to workplace, quality of service, low cost, referral although they still had difficulty in accessing the facility and this was significant (p < 0.001). Self-reported history of being stigmatized (irrespective of the person or group who stigmatized them) is also associated with difficulty in accessing the facility (p = 0.003) (see Table 2).

**Table 2:** Association of Social related factors and accessibility to facilities

	Accessibility to facility		Total	p
	Difficult	Easy		
Prior LTFU				
Yes	78 (82.98)	16 (17.02)	94 (31.97)	*0.031* (*LR*)
No	135 (69.23)	60 (30.77)	195 (66.33)	
Missing	3 (60.00)	2 (40.00)	5 (1.70)	
Inaccessibility factors				
Bad/risky roads	17 (100.00)	0 (0)	17 (5.78)	< *0.001*
Cost of transportation	71 (100.00)	0 (0)	71 (24.15)	
Easy access	0 (0)	78 (100.00)	78 (26.53)	
Far	73 (100.00)	0 (0)	73 (24.83)	
Working hours	55 (100.00)	0 (0)	55 (18.71)	
Reason for choosing facility				
Availability of female provider	4 (100.00)	0 (0)	4 (1.36)	< *0.001*
Availability of drugs	32 (71.11)	13 (28.89)	45 (15.31)	
Facility is only available option	25 (92.59)	2 (7.41)	27 (9.18)	
Low cost	28 (90.32)	3 (9.68)	31 (10.54)	
Recommended/referral	11 (100.00)	0 (0)	11 (3.74)	
Timeliness/promptness of service	9 (100.00)	0 (0)	9 (3.06)	
Proximity	51 (57.95)	37 (42.05)	88 (29.93)	
Trust in provider/quality of service	45 (68.18)	21 (31.82)	66 (22.45)	
Others	11 (84.62)	2 (15.38)	13 (4.42)	
History of being stigmatized				
Yes	90 (82.57)	19 (17.43)	109 (37.07)	*0.003*
No	121 (67.22)	59 (32.78)	180 (61.22)	
Missing	5 (100.00)	0 (0)	5 (1.70)	
Stigmatised by whom				
Community/neighbour	28 (93.33)	2 (6.67)	30 (30.61)	0.127
Family	23 (88.46)	3 (11.54)	26 (26.53)	
Friend	13 (68.42)	6 (31.58)	19 (19.39)	
HCW	7 (70.00)	3 (30.00)	10 (10.20)	
Spouse/partner	10 (76.92)	3 (23.08)	13 (13.27)	

*LR* likelihood ratio, *p* p-value

Italicized is significant at < 0.05

Lack of support (p < 0.001), particularly psychological/emotional (p < 0.001) and financial support (p < 0.001) were strongly associated with difficulty in accessing health care facility. Not perceiving adequate family support (p = 0.02) and not perceiving adequate support from health care workers (p = 0.02) were also associated with difficulty in accessing health facility (see Table 3).

**Table 3:** Association of other social related factors and accessibility to facilities

	Accessibility to facility		Total	p
	Difficult	Easy		
Know partner status				
Yes	152 (72.38)	58 (27.62)	210 (71.43)	0.728
No	45 (77.59)	13 (22.41)	58 (19.73)	
Missing	19 (73.08)	7 (26.92)	26 (8.84)	
Partner status				
Positive	93 (73.23)	34 (26.77)	127 (43.20)	0.754
Negative	59 (71.08)	24 (28.92)	83 (28.23)	
Missing	64 (76.19)	20 (23.81)	84 (28.57)	
Disclosed HIV status				
Yes	152 (72.38)	58 (27.62)	210 (71.48)	0.728
No	45 (77.59)	13 (22.41)	58 (19.73)	
Missing	19 (73.08)	7 (26.92)	26 (8.84)	
Lack support				
Yes	179 (75.21)	59 (24.79)	238 (80.95)	*<* *0.001*
No	16 (48.48)	17 (51.52)	33 (11.22)	
Missing	21 (91.30)	2 (8.70)	23 (7.82)	
Type of lack of support				
Financial	126 (70.79)	52 (29.21)	178 (60.54)	*<* *0.001*
Psychological/emotional	53 (88.33)	7 (11.67)	60 (20.41)	
Others (disclosure to spouse/knowledge of illness…)	16 (48.48)	17 (51.52)	33 (11.22)	
Missing	21 (91.30)	2 (8.70)	23 (7.82)	
Perception of adequate family support				
Yes	165 (70.21)	70 (29.79)	235 (79.93)	*0.028* (*LR*)
No	46 (86.79)	7 (13.21)	53 (18.03)	
Missing	5 (83.33)	1 (16.67)	6 (2.04)	
Perception of adequate spousal support				
Yes	157 (72.02)	61 (27.98)	218 (74.15)	0.495 (LR)
No	48 (76.19)	15 (23.81)	63 (21.43)	
Missing	11 (84.62)	2 (15.38)	13 (4.42)	
Perception of adequate support from healthcare workers				
Yes	174 (70.45)	73 (29.55)	247 (84.01)	*0.014* (*LR*)
No	36 (90.00)	4 (10.00)	40 (13.61)	
Missing	6 (85.71)	1 (14.29)	7 (2.38)	

*LR* likelihood ratio, *p* p-value

Italicized is significant at < 0.05

In the multivariate analysis of predictors of accessibility to facilities (Table 4), those who reported difficult access to health care facilities were twice as likely to have had a prior LTFU (OR 2.5 [95% CI 1.3–4.8], p = 0.008), twice more likely to have had a history of being stigmatized (OR 2.1 [95% CI 1.1–3.8], p = 0.02), about three times more likely to have lacked support (OR 2.8 [95% CI 1.3–6.0], p = 0.01) and about four times more likely to have perceived inadequate HCW support (OR 3.8 [95% CI 1.2–11.2], p = 0.02).

**Table 4:** Multivariate analysis of predictors of accessibility to facilities

n = 266	OR	CI	p
Predictors			
Prior LTFU			
No	Ref		
Yes	2.456	1.258–4.796	*0.008*
History of stigma			
No	Ref		
Yes	2.050	1.099–3.822	*0.024*
Lack of support			
No	Ref		
Yes	2.765	1.272–6.012	*0.010*
Age (years)			
≤ 35	Ref		
≥ 36	0.981	0.549–1.752	0.949
Perception of adequate HCW support			
Yes	Ref		
No	3.717	1.232–11.213	*0.020*

*OR* odds ratio, *CI* confidence interval, *p* p-value, *Ref* reference value set at 1

Italicized is significant at < 0.05

## Discussion

This study showed that 38% of clients tracked were LTFU indicating that this phenomenon is still a common programmatic challenge [8] as exemplified in previous studies [1–7]. However, about 7.5% of patients tracked with the support of NEPWHAN were returned to care through active tracking and engagement. About half of returnee PLHIV reported that access to health facility was difficult, just about a third (28.8%) had a previous LTFU event while about a quarter of them (23.5%), reported experiencing or suspecting stigmatizing behavior. This research showed that bad/dangerous roads, cost of transportation, far distance to facility also depicted in other studies [10, 23, 24] and long working hours are all strongly associated with difficulty in accessing health facilities. Surprisingly, most of the clients chose to use these facilities because of the quality of service offered, easy accessibility or due to the affordability of its services. Self-reported history of being stigmatized is associated with difficulty in accessing the facilities.

Support from the family was robust for 82.7% respondents, but lack of support, particularly psychological/emotional (88.3%) and financial support (70.8%) is strongly associated with difficulty in accessing health care facility. Not perceiving adequate family support and not perceiving adequate support from health care workers were also associated with difficulty in accessing health facility. Those who reported difficult access to health care facilities were twice as likely to have had a prior LTFU, twice more likely to have had a history of being stigmatized, about three times more likely to have lacked support and about four times more likely to have perceived in-adequate HCW support.

Our study has demonstrated that untoward psycho-social realities of PLHIV exist including lack of adequate social, financial and health provider support, difficult access to the facilities and perceived stigma which have been posited in other studies [15, 16, 20, 25]. These issues impact negatively on the abilities of PLHIV to remain in care or treatment, adhere to visits or medications as shown in previous studies [15, 17, 18]. It also showed that socio-structural realities interact extensively with health systems gaps to impede linkage of HIV infected individuals to treatment amplifying poor clinical and epidemiologic outcomes [9] that may affect HIV program effectiveness and efficiency [20, 21, 26].

Social and economic dimensions such as cost of accessing care, relationships, disclosure issues and social support by family, partner, community and health care workers are some of the barriers to retention in HIV care which must be highly considered by service providers if we must cut off on the increasing attrition because of LTFU among PLHIV and meet up the 2nd 90 of the UNAIDS targets.

This study has some limitations because it is based on self-report of previous events which might be subject to recall and report bias while the sample of patients who returned to care might be fundamentally different from who refused to return to care. Potential reasons why some of the patients tracked refused to return to care could be beyond social issues and may be related to unavailability or inadequate attention to facility and time-dependent programmatic factors like patient–provider relationship, staffing issues, early adherence patterns, CD4^+^ cells count, unsuppressed viral loads [6, 25] that needs to be addressed in all HIV programs.

There is an urgent need to build social constructs and dimensions into HIV service delivery models to improve retention in care of PLHIV. Such constructs would include home visits, community-based care services, transportation subsidies and robust social support systems which have been demonstrated in previous studies to reduce the likelihood of LTFU [26, 27]. The authors advocate for further studies on how different care models impact on retention of patients in care and reasons reported by patients with previous history of LFTU that is consistent with current occurrence.

## Data Availability

The datasets generated and/or analysed during the current study are not publicly available due to institutional policy but are available from the corresponding authors on reasonable request.

## Abbreviations

AIDS: acquired immuno-deficiency syndrome
ART: anti-retroviral therapy
ARV: anti-retroviral
CDC: Centre for Disease Control
CI: confidence interval
EMRS: electronic medical records system
HIV: human immune-deficiency virus
IHVN: Institute of Human Virology Nigeria
LMIC: low and middle-income countries
LTFU: loss to follow-up
NEPWHAN: Network of People Living with HIV and AIDS in Nigeria
NHREC: National Health Research Ethics Committee
OR: odds ratio
PEPFAR: President’s Emergency Plan for AIDS Relief
PLHIV: people living with human immunodeficiency virus
P: p-value
SSA: sub-Saharan Africa
SPSS. 20: IBM Corp. Released 2011. IBM SPSS Statistics for Windows, Version 20.0
US: United States
